# Predicting partition coefficients for the SAMPL7 physical property challenge using the ClassicalGSG method

**DOI:** 10.1007/s10822-021-00400-x

**Published:** 2021-06-28

**Authors:** Nazanin Donyapour, Alex Dickson

**Affiliations:** Department of Computational Mathematics, Science and Engineering, Michigan State University, East Lansing, Michigan, USA; Department of Computational Mathematics, Science and Engineering, Michigan State University, East Lansing, Michigan, USA; Department of Biochemistry and Molecular Biology, Michigan State University, East Lansing, Michigan, USA

**Keywords:** SAMPL7 log *P* challenge, Geometric scattering for graphs, Neural networks, Partition coefficient, molecular representations, log P, machine learning, chemical features

## Abstract

The prediction of log *P* values is one part of the statistical assessment of the modeling of proteins and ligands (SAMPL) blind challenges. Here, we use a molecular graph representation method called Geometric Scattering for Graphs (GSG) to transform atomic attributes to molecular features. The atomic attributes used here are parameters from classical molecular force fields including partial charges and Lennard-Jones interaction parameters. The molecular features from GSG are used as inputs to neural networks that are trained using a “master” dataset comprised of over 41, 000 unique log *P* values. The specific molecular targets in the SAMPL7 log *P* prediction challenge were unique in that they all contained a sulfonyl moeity. This motivated a set of ClassicalGSG submissions where predictors were trained on different subsets of the master dataset that are filtered according to chemical types and/or the presence of the sulfonyl moeity. We find that our ranked prediction obtained 5th place with an RMSE of 0.77 log *P* units and an MAE of 0.62, while one of our non-ranked predictions achieved first place among all submissions with an RMSE of 0.55 and an MAE of 0.44. After the conclusion of the challenge we also examined the performance of open-source force field parameters that allow for an end-to-end log *P* predictor model: General AMBER Force Field (GAFF), Universal Force Field (UFF), Merck Molecular Force Field 94 (MMFF94) and Ghemical. We find that ClassicalGSG models trained with atomic attributes from MMFF94 can yield more accurate predictions compared to those trained with CGenFF atomic attributes.

## 1 Introduction

The logarithm of the octanol-water partition coefficient (*P*) of a neutral compound is referred to as log *P* and can also be denoted as log *K*_*ow*_ or log *P*_*ow*_. The partition coefficient itself is defined as the ratio of the concentrations of a compound in a two-phase system in equilibrium. One of the main applications of log *P* is in drug design and discovery. It is a quantitative descriptor of lipophilicity, which affects the absorption, distribution, metabolism, elimination, and toxicology (ADMET) of a drug compound in the body. Additionally, the log *P* value of a chemical compound determines its drug-likeness and is included in the famous Lipinski’s Rule of Five [1]. The applications of log *P* are not specific to drug design and extend to other fields such as agriculture [2–4], environmental science [5–7] among many others.

Considering the widespread usage of log *P* and the cost associated with experimental measurements, a large variety of computational methods such as XlogP3 [8], AlogP [9], ClogP [10], KowWIN [11], JPlogP [12] László et al [13], Huuskonen et al [14], MlogP [15], iLogP [16], Manhold [17], AlogPS [18], S+logP [19], CSLogP [20], Silicos-IT LogP [21], TopP-S [22], OpenChem [23] have been developed over the years. These methods employ various techniques and algorithms for predicting log *P* and have their pros and cons as explained in our previous work [24]. In publications, these methods often use their own specific test sets, making the comparison between different algorithms challenging. Hence, benchmarks and standardized test sets are needed to effectively compare these methods and further advance the research on log *P* prediction.

To help meet this need, the statistical assessment of the modeling of proteins and ligands (SAMPL) [25] project recently created a distinct blind challenge for predicting log *P* allowing fair evaluation and comparison of different log *P* prediction methods (SAMPL6 in 2019 [26] and SAMPL7 in 2020 [27]). In this challenge, the participants predict log *P* for a set of drug-like molecules and the predictions are assessed using experimental log *P* values that are revealed later. The submitted prediction methods are classified into one of the following categories: Empirical methods, Physical molecular mechanics (MM)-based, Physical quantum mechanics (QM)-based, or Mixed methods. Empirical methods [8–22, 24, 28, 29] are data-driven methods in which predictor models are trained directly on a dataset of molecules. The empirical category includes methods that employ atomic/fragment-based additive methods, machine learning, and quantitative structure-property relationship (QSPR) approaches. In MM-based methods [30, 30–34], molecular dynamics simulations are run and used to estimate the solvation free energy. Then, the log *P* for a compound is calculated analytically from the solvation energy. QM-based methods [35–41] utilize the solvation free energy estimated from the quantum mechanical energy calculations. The mixed approaches [40, 42–44] employ the combination of physical (QM/MM-based) and empirical techniques.

The main advantage of empirical methods is that they are quite fast compared to physical (MM/QM-based) methods. However, training a log *P* predictor model with the ability to generalize to new data is not easy. For example, the Root Mean Squared Error (RMSE) of the best log *P* predictor model for the NonStar [17] test set is 0.82 [22], which is higher than expected experimental sources of error, even taking into account different experimental methods for log *P* measurement [45]. This test set, which is publicly available [46] has 43 compounds that are unlike compounds typically found in the training sets of tested methods (see Table 7 in [22]). Similarly, the SAMPL7 challenge [27] involves a set of 22 molecules that each contain a sulfonyl moiety, which is relatively under-represented in training sets. Here we examine the accuracy of different training sets in predicting log *P* values for compounds with special structures. A master training dataset which includes 41, 409 molecules, was filtered according to chemical elements and/or the existence of the sulfonyl moiety to generate three smaller datasets.

On the other hand, the performance of the empirical models also depends on the choice of molecular features used for training the models. Generally, molecular features are a set of numerical values that describe the relevant properties of a molecule. Additive empirical methods such as XlogP3 [8], Alog [9], ClogP [10], KowWIN [11], JPlogP [12] simply construct a vector of atomicor fragment-based attributes and predict log *P* using a function that sums contributions from each of the component attributes. Additive methods are inherently approximate, as they do not take into account the entire structure of a molecule. Other methods address this through a combination of molecular and atomic- or fragment-based descriptors [21, 22]. The challenge we seek to address in our approach is to develop a set of molecular features that succinctly describe the contributions of each atom while taking the molecular structure into account.

A natural way to represent molecules is to use a graph where nodes are atoms and the edges are bonds. Graph representation of molecules is becoming very popular in recent years, and it enables us to represent the complex molecular structures effectively and subsequently ensure high performance of models [23, 47–52]. We should notice that graph-based models are naturally invariant to translation, rotation, and reflection symmetries. To ensure symmetry with respect to re-indexation of atoms, methods such as convolution neural networks and a recently developed graph data analysis method called geometric scattering for graphs (GSG) [53] can be used.

Our approach in this work for predicting log *P* is based on a graph representation of molecules that employs GSG for generating invariant molecular features from atomic attributes. GSG is beneficial in that it uses a fast analytical method for creating molecular features. The molecular features are of equal length for molecules with any number of atoms allowing us to use any distance metric for calculating the similarity of two molecules. Here, we use atomic attributes taken from molecular mechanics force fields including partial charges, atom type, and Lennard-Jones interaction parameters: radius (*r*) and well-depth (*ϵ*). The GSG molecular features are used as inputs to neural networks that are trained to predict log *P*. We refer to this combined approach as “ClassicalGSG”.

In our previous work [24], we employed ClassicalGSG to examine the performance of two force field parameter matching programs: CHARMM General Force Field (CGenFF) [54, 55] and General AMBER Force Field 2 (GAFF2) [56, 57]. The NN models were trained using a dataset of molecules made available by OpenChem [23] and we showed that CGenFF-generated parameters with a specific ad hoc scheme of classifying CGenFF atomic types achieved the highest accuracy in predicting log *P* values.

For the SAMPL7 target molecules we used the best performing parameter sets to train four log *P* predictor models. One of our verified (but non-ranked) prediction sets achieved the lowest RMSE (0.55) and the second-lowest Mean Absolute Error (MAE) of 0.44 among all the submitted predictions. We note that in SAMPL6 [26] the best set of predictions was using Cosmotherm [36], achieving an RMSE of 0.38, and 10 models achieved an RMSE of less than 0.5. In SAMPL7 none of the submitted predictions were below this threshold, implying that these molecules had specific structures that introduced difficulty into both the empirical and physical predictions.

In this work we describe the process of curating the four training datasets, training the models and making predictions for SAMPL7 target molecules. Further, to achieve better predictions we examined the performance of various open-source force fields such as General AMBER Force Field (GAFF) [58], Universal Force Field (UFF) [59], Merck Molecular Force Field 94 (MMFF94) [60, 61] and Ghemical [62]. Our results show that MMFF94 models create predictors that on average are as accurate or better than those created with CGenFF. We conclude with a discussion regarding the curation of training sets for the SAMPL7 challenge, the performance of open-source force field generator tools and the code available in the ClassicalGSG package.

## 2 Methods

### SAMPL7 log *P* challenge molecules and curation of the training datasets

**Fig. 1:**
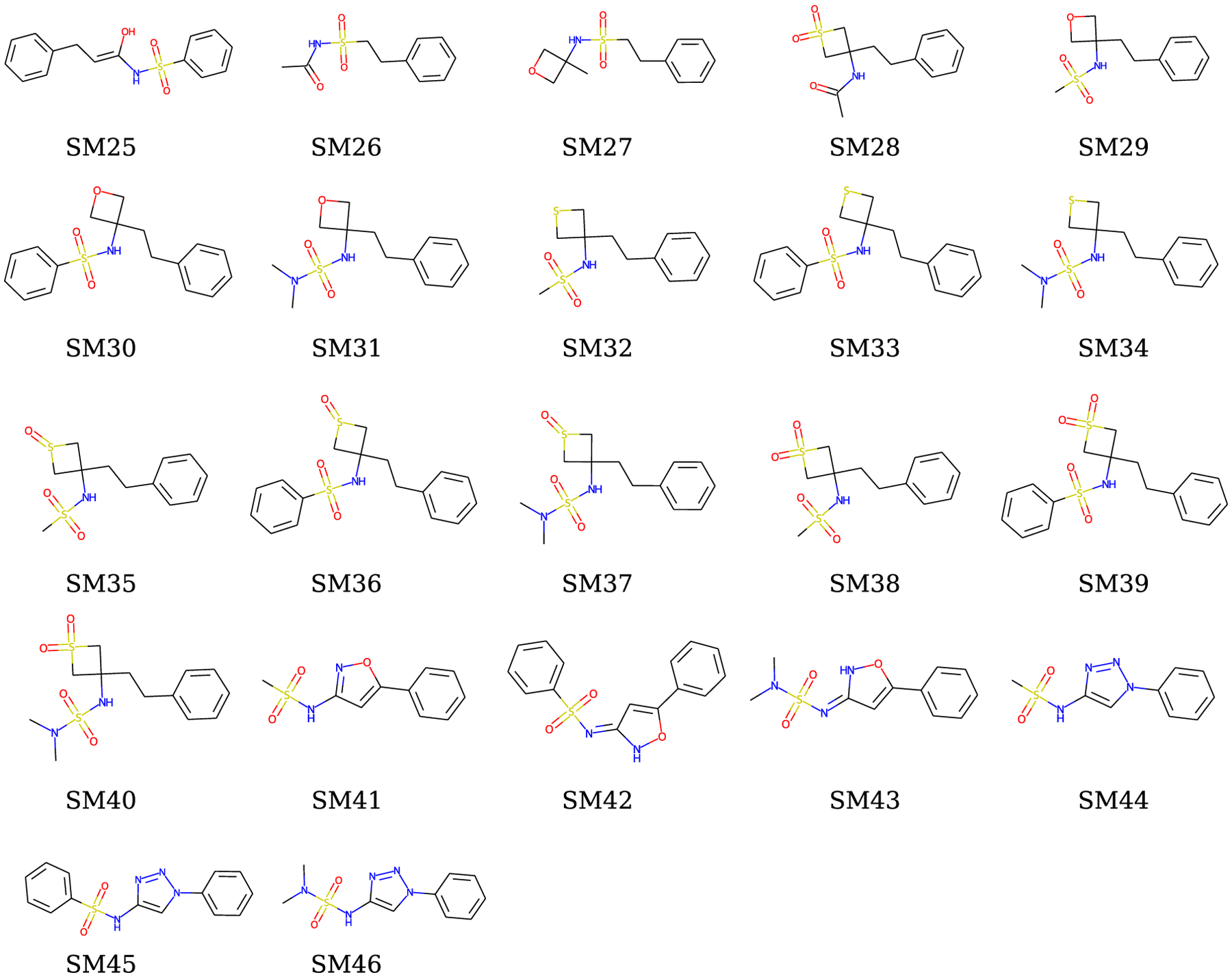
The SAMPL7 log *P* challenge molecules. The SAMPL7 target molecules are shown in their 2D structures in their neutral microstate (micro000). The 2D structures are generated and drawn from SMILES by RDkit [64].

The SAMPL7 target molecules were synthesized by the Ballatore group at the UC San Diego university and their log *P* values were measured experimentally [63]. This collection includes 22 small drug-like molecules whose 2D structures in their neutral state are shown in Figure 1. These molecules all consist of only five atomic elements of (C, N, O, S, and H) and all have a sulfonyl moiety. The molecular weights vary from 227.285 to 365.476.

For the SAMPL7 challenge, we first built a master training dataset by combining the log *P* datasets in Table 1. The physical properties database (PHYSPROP) [65] was built by the Syracuse Research Corporation (SRC) and contains the log *P* values of over 41, 000 diverse chemical compounds. Here, we used the public version of PHYSPROP. The Huuskonen dataset [14] has 1, 844 unique molecules, combining 326 molecules from its initial version [66] with 1, 663 molecules from the Klopman dataset [67]. The 1, 844 molecules in the Huuskonen dataset have been organized into a training set with 1, 496 compounds and a test set with 348 compounds. Here we use molecules from the Huuskonen training set. The TopP-S dataset consists of 8, 199 chemical compounds, initially compiled by Hansch et al. [68] and then compiled by Cheng et al. [8] to include only molecules with reliable experimental log *P* values. The OpenChem dataset was curated from the PHYSPROP drug database [23] and contains of 14, 176 molecules. The Logpt_all_data_training, ALOGPS_3_01, and Logpt_challenge_training are public log *P* training sets which can be downloaded from https://ochem.eu. The RDkit program [64] is employed to create canonical SMILES for molecules in these 7 datasets. After removing duplicate molecules, 44, 595 molecules remained in the dataset. As the generation of CGenFF atomic attributes failed for some molecules, we ended up with 41, 409 molecules in our dataset, which we refer to as the “master dataset.”

The master dataset itself serves as “DB1”, which is used to train a GSG model to generate a set of predictions for the SAMPL7 molecules. The presence of only five atomic elements C, N, O, S, and H in the SAMPL7 target molecules motivated us to make a subset of the master dataset where each compound has only has these atomic elements, which we call “DB2”. Molecules that either had another element not listed above or did not have the full set of elements were not selected. The DB2 dataset has 3, 482 molecules. Also, the existence of a specific structure - a sulfonyl moiety - in all of the SAMPL7 target molecules inspired us to generate the third dataset by filtering the master training set and keeping only those with sulfonyl moiety. The “HasSubstructMatch” function of RDKit was used to check if a molecule has this moiety. The obtained training dataset is referred to as “DB3” and has 2, 379 molecules. The fourth training set was obtained by filtering the master dataset and keeping only those with both a sulfonyl moiety and the following elements (C, N, O, S, and H). This training set has 1, 482 molecules, and we refer to it as “DB4”. These four datasets DB1, DB2, DB3, and DB4, are used to train four ClassicalGSG models and generate four sets of predictions for the SAMPL7 target molecules.

Further, to assess the performance of the open-source force field tools we use a group of external test sets including FDA [8], Star [17], NonStar[17] and Huuskonen [14, 66] and SAMPL6 molecules set [26] (see Table 2 in Refs [24]). To quantify our uncertainty, we chose molecules from these test sets that are similar to the set of SAMPL7 molecules. More specifically, these test sets are filtered to include molecules with a sulfonyl moiety and to include each of the elements (C, N, O, S, and H). Molecules that contained other elements were excluded. The selected 44 molecules are filtered further by keeping molecules which their molecular weight is in the range of SAMPL7 molecules weights. The resulting test set has 36 molecules and referred to as S7_TEST.

### Generating atomic attributes

In the Geometric Scattering for Graphs method [53], molecular features are generated by “scattering” atomic attributes over the graph structure of the molecule. Here our set of atomic attributes includes partial charges, atom type, and Lennard-Jones interaction parameters for the atoms of each molecule. These atomic attributes are generated either by CGenFF [54, 55] or open-source force fields such as GAFF [58], UFF [59], MMFF94 [60, 61] and Ghemical [62].

To generate CGenFF atomic attributes, OpenBabel [69, 70] is used to generate 3D structures for the molecules from SMILES and save them in mol2 format. The mol2 file is passed to the CGenFF tool of the SilcsBio package (http://silcsbio.com) to create a CGenFF parameter file in str format. Atomic partial charges and atomic types for each atom are extracted from the str file. Then two Lennard-Jones parameters – radius (*r*) and well-depth (*ϵ*) – are extracted from CHARMM parameter file (par_all36_cgenff.prm) for each atom type in the molecule. The one-hot encoding format is used for representing atomic types while atomic charge and two Lennard-Jones parameters are scalar values. CGenFF has 169 atomic types, and to reduce the number of atomic type categories, as in our previous work [24], we manually grouped CGenFF atom types into 36 groups and refer to this as Atom Category 36 “AC36” (see Table S1 [24]).

All of the open-source force fields such as GAFF, UFF, MMFF94, and Ghemical are implemented inside OpenBabel and making it easier to generate force field parameters for a molecule. The SMILES is used to generate an OpenBabel molecule and 3D structures. Using the “Setup” function of the force field method the atomic parameters are generated for a given molecule. This method is straight forward and does not require any external program. We use all the atomic types generated by each of these force fields without further grouping them, which we denote as “ACall”.

### Geometric scattering for graphs

Geometric scattering for graphs (GSG) [53] is a non-trainable graph feature extraction method proposed by Gao et al. [53] that is analogous to Graph Convolution Neural Networks (GCNs) [71]. Unlike the GCNs, GSG uses a cascade of designed wavelet filters instead of convolution filters with learned parameters. Another advantage of GSG is that its features can be directly assigned to particular atomic attributes, whereas this analysis in GCNs is more challenging. The GSG method has been shown to be a powerful tool for representing the graph structures in varied datasets including the classification of enzymes via protein structural features [53]. Here, we use GSG to generate invariant features from the graph representation of small organic molecules. Each atom is represented by a node and the edges are covalent bonds. A vector of attributes is associated with each atom, which can include the atomic number or more specific atomic types. GSG encodes the geometric information of molecules in an adjacency matrix and generates wavelet filters to capture several convolutions of node attributes that take into account the graph structure of the molecule. The architecture of GSG is shown in Fig 1 of Ref. [24].

Here we describe of the mathematical construction of this method (additional discussion can be found in Refs. [24, 53]). Let *G* = (*V, E, W*) be a weighted graph where *V* is the set of nodes, and *E* is the set of edges in the graph. A signal function $x\left(v_{i}\right)\to {\mathbb{R}}^{N}$ is defined on each node where *N* is the number of node attributes, 1 *< i* ≤ *n* is the index of a node, and *n* is the number of nodes in the graph. GSG uses a lazy random walk matrix, defined as follows

(1) $$P=\frac{1}{2}\left(I+AD^{-1}\right)$$

where *I* is the identity matrix, *A* is the adjacency matrix showing the node connectivity, and *D* is the degree matrix. The lazy random walk includes self-connections and acts like a Markov process with a transition matrix of *AD*^−1^. Higher powers of *P* (e.g. *P*^*t*^) represent the probability distribution of a graph lazy random walk after *t* steps. Here, this can be seen as a random walk over the structure of the molecule, where “steps” are transitions between atoms. These are used to create a set of wavelet matrices, denoted *Ψ*_*j*_, where

(2) $$\Psi_{j}=P^{2^{j-1}}-P^{2^{j}}$$

The wavelet matrices are thus convolution-like filters, used to transform the information of nodes at different scales and are also referred to as graph wavelet transforms. These are applied to graph signals **x** to generate geometric scattering transforms, which are defined at three orders (zeroth, first and second) that are named based on the number of transformations *Ψ*_*j*_ applied to **x**.

The zeroth order scattering moments (**S**_0_) are the untransformed *q*^th^ moments of **x**, defined as follows:

(3) $$S_{0}=\sum_{i=1}^{n}x{\left(v_{i}\right)}^{q},\;1\leq q\leq Q$$

where *Q* is the number of moments considered for each signal in **x**. The number of features in **S**_0_ is equal to *NQ*. The **S**_0_ are the simplest invariant features but cannot capture the variability of **x** completely. Hence, the higher order scattering is defined that takes into account the molecular structure.

The first order scattering moments (**S**_1_) are *q*^th^ order moments of **x** “scattered” by the wavelet matrices *Ψ*_*j*_:

(4) $$S_{1}=\sum_{i=1}^{n}{\left|\Psi_{j}x\left(v_{i}\right)\right|}^{q},\;1\leq j\leq J\;1\leq q\leq Q$$

where *J* is the maximum wavelet scale, and the total number of first order features is equal to *NJQ*.

The second order scattering moments (**S**_2_) are constructed by applying wavelet matrices $\Psi_{j^{\prime }}$ to |*Ψ*_*j*_**x**(*v*_*l*_)| at different scales (e.g. where *j* ≠ *j*′):

(5) $$S_{2}=\sum_{i=1}^{n}\left|\Psi_{j^{\prime }}\right|\Psi_{j}x\left(v_{i}\right){||}^{q},\;1\leq j<j^{\prime }\leq J\;1\leq q\leq Q$$

**S**_2_ combines wavelet transforms at two scales 2^*j*^ and $2^{j^{\prime }}$ and generates features that connect the patterns of short- and long-range subgraphs within the full graph. There are a total of $\frac{1}{2}NJ(J-1)Q$ second order features.

The stack of {**S**_0_, **S**_1_, **S**_2_} generates symmetry-invariant and informative information for a given molecule. Note that GSG generates features with the same length regardless of the size of the molecule, allowing us to use any distance metric for similarity measurements. Here, the adjacency matrices are constructed from the 2D structure of molecules. If there is a bond between nodes *i* and *j*, *A*_*ij*_ is set to 1, and is 0 otherwise.

### Neural network architecture

The neural networks we employed for training the ClassicalGSG models are multilayer perceptron (MLP) networks and we implemented them using the PyTorch package [72]. We used Rectified Linear Unit (ReLU) as the nonlinear activation functions in our models. To tune the hyperparameters and train the models, we performed a 5-fold cross validation using Skorch [73] where we did a comprehensive grid search in the space of hyperparameters to find the best performing models. We used the MSELOSS (Mean Squared Error) and Adam (Adaptive Momentum Estimation)[74] as the loss function and optimizer of the parameters, respectively. We chose an adaptive learning rate policy with the initial value of 0.005 which drops by a factor of 0.5 every 15 steps. The “standardization” function from the scikit-learn package [75] was used for regularizing molecular features. The hyperparameters and other parameters of NNs are summarized in Table 2.

## 3 Results

### Uncertainty estimations

Upon submission of our results to the SAMPL7 organizers, our predictions for uncertainty in log *P* were simply the standard errors of the mean of the predictions using the four training sets. Here we first calculate more accurate estimates for the prediction uncertainty using prediction intervals (PIs) obtained separately for each of the four predictors. PIs define the range of values in which predictions for new data are expected to lie with a defined probability. For example, a 90% PI in the range of [*a, b*] indicates that a future prediction will fall into the range [*a, b*] 90% of the time. There are a variety of methods for calculating PIs for NNs, such as bootstrapping [76, 77], Mean-Variance Estimation (MVE) [78], Delta, and Bayesian methods. In this paper, we utilize a parametric approach similar to the MVE method. However, unlike the MVE, our method constructs PIs from the Mean Absolute Errors (MAE) between the predicted and observed values of similar inputs rather than all of the input data. The PIs are determined by finding a MAE value (*ϵ*_90_) below which 90% of MAE values fall. Thus for a future prediction $\left(\hat{y}\right)$ the PI is defined as $\left[\hat{y}-\epsilon_{90},\hat{y}+\epsilon_{90}\right]$.

**Fig. 2:**
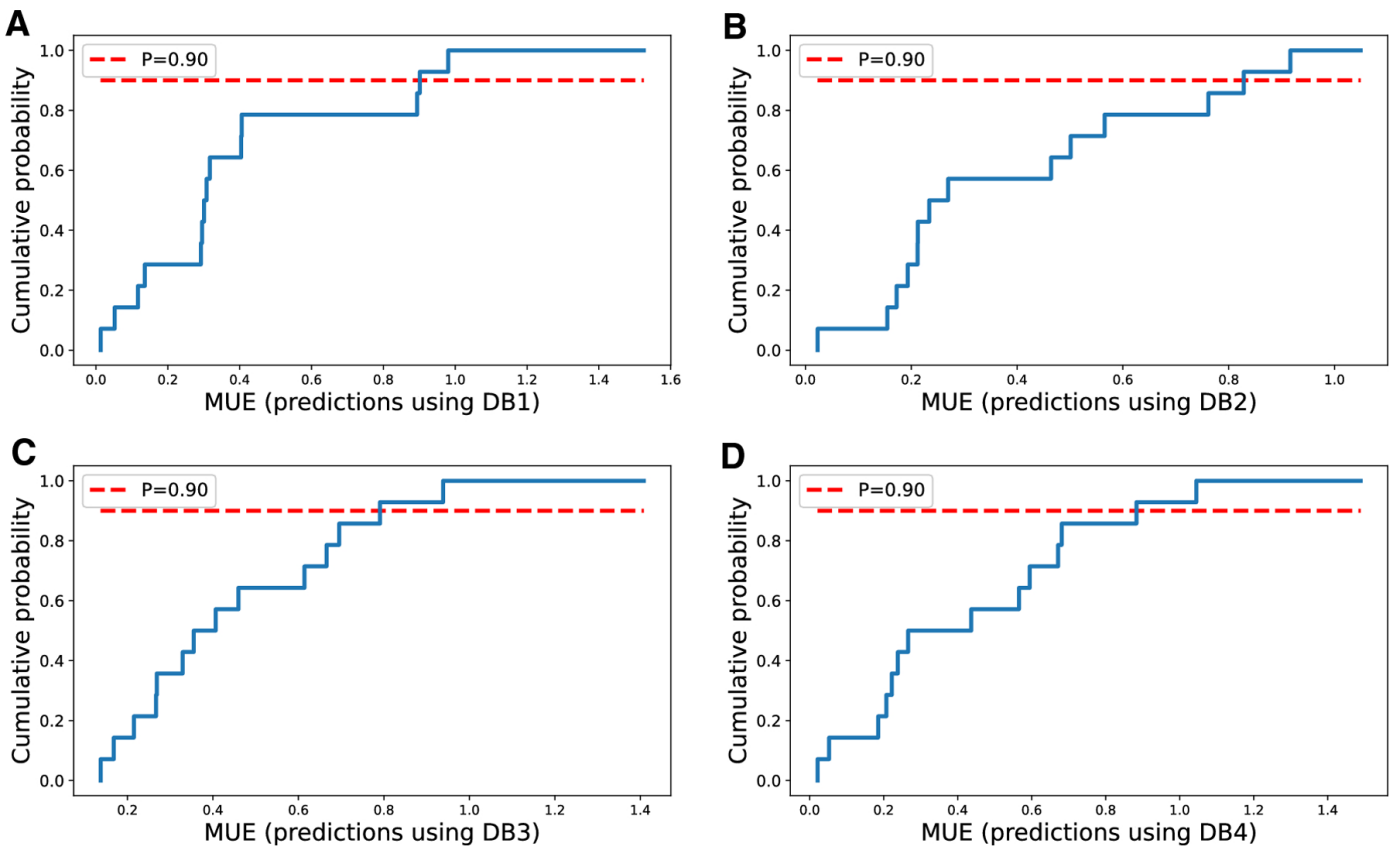
Cumulative distribution of MAE of molecules in the S7_TEST set. The solid blue line shows the cumulative distributions for each set of predictions. The dashed red line represents the probability of 90%. Panels A through D show MAEs using models trained on DB1 through DB4, respectively.

To construct PIs, we make four sets of predictions for the S7_TEST dataset using ClassicalGSG models trained on DB1, DB2, DB3, or DB4 training sets. As mentioned in Section 2, S7_TEST is a subset of external test sets containing molecules similar to the SAMPL7 molecules. For training these models, we used the parameters of the best ClassicalGSG models obtained in our previous work [24]. More specifically, atomic attributes from CGenFF parameters, 2D molecular structure information and AC36 atomic types fed to GSG with parameters of maximum wavelet number (*J*) of 4, and all scattering operators (zeroth, first, and second order) to generate molecular features. After making predictions using these models, the MAE values are calculated for S7_TEST for each set of predictions. We then binned them in a histogram with 20 bins to determine cumulative probability distributions shown in Figure 2.

**Fig. 3:**
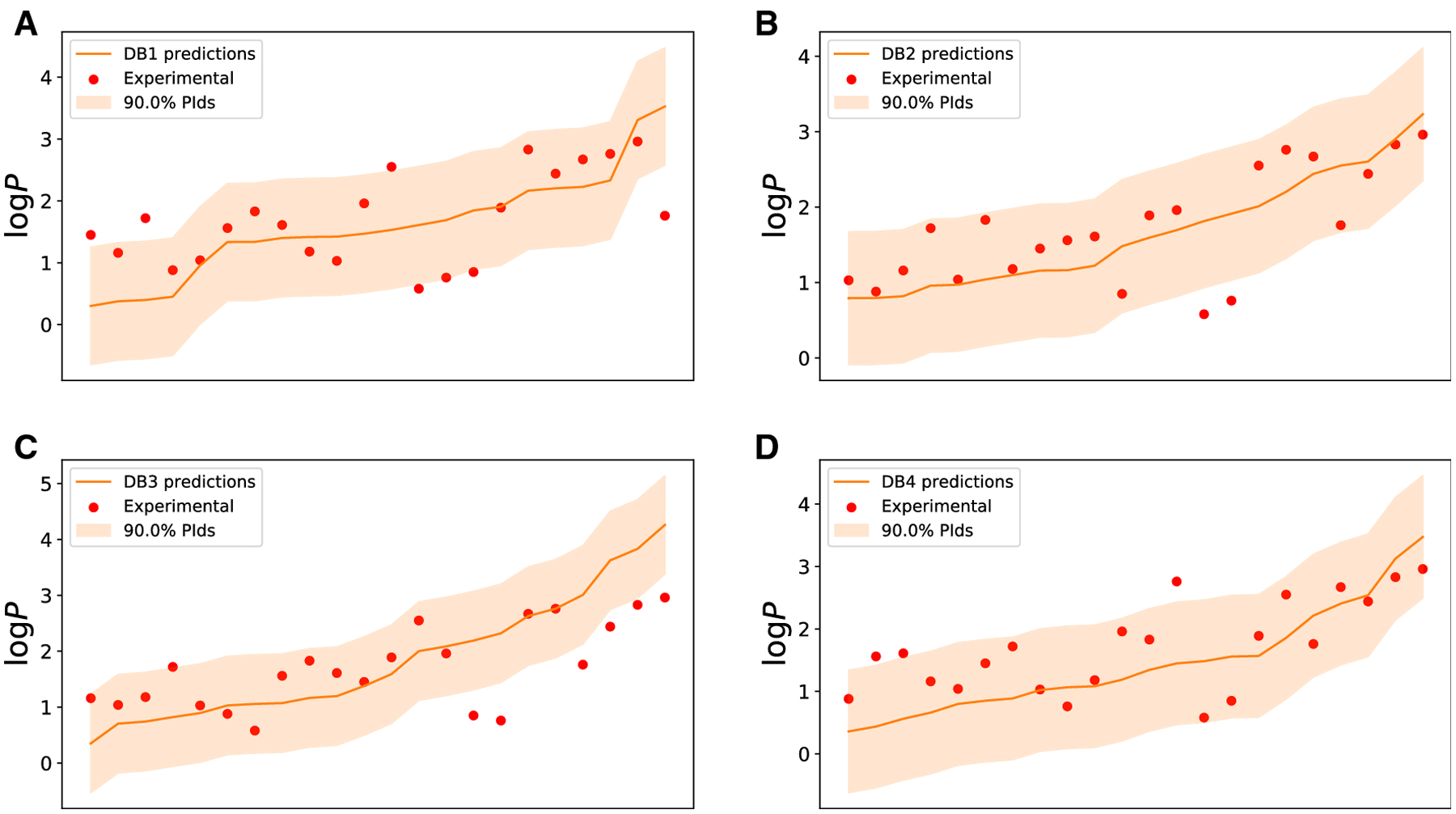
Prediction intervals of log *P* predictions for the SAMPL7 molecules. The experimental log *P* values are shown in red circles as a scatter plot. The predictions are shown in a red line, and the orange wide range shows the prediction intervals (PIs). Panels A through D show predictions from models trained on DB1 through DB4, respectively. In all cases, data is sorted according to the predicted log *P* values.

The four sets of predictions made for SAMPL7 target molecules using each model and their 90% PIs are shown in Figure 3. We determined the coverage of PIs for experimental log *P* values for each model and the results are shown in Table 3. The ClassicalGSG_DB2 method has the highest coverage of 90.90%, as expected, although other predictors fall below this threshold. This could indicate that the SAMPL7 log *P* values were more difficult to predict than the similar S7_TEST molecules.

### Predictions for SAMPL7 log *P* challenge

**Fig. 4:**
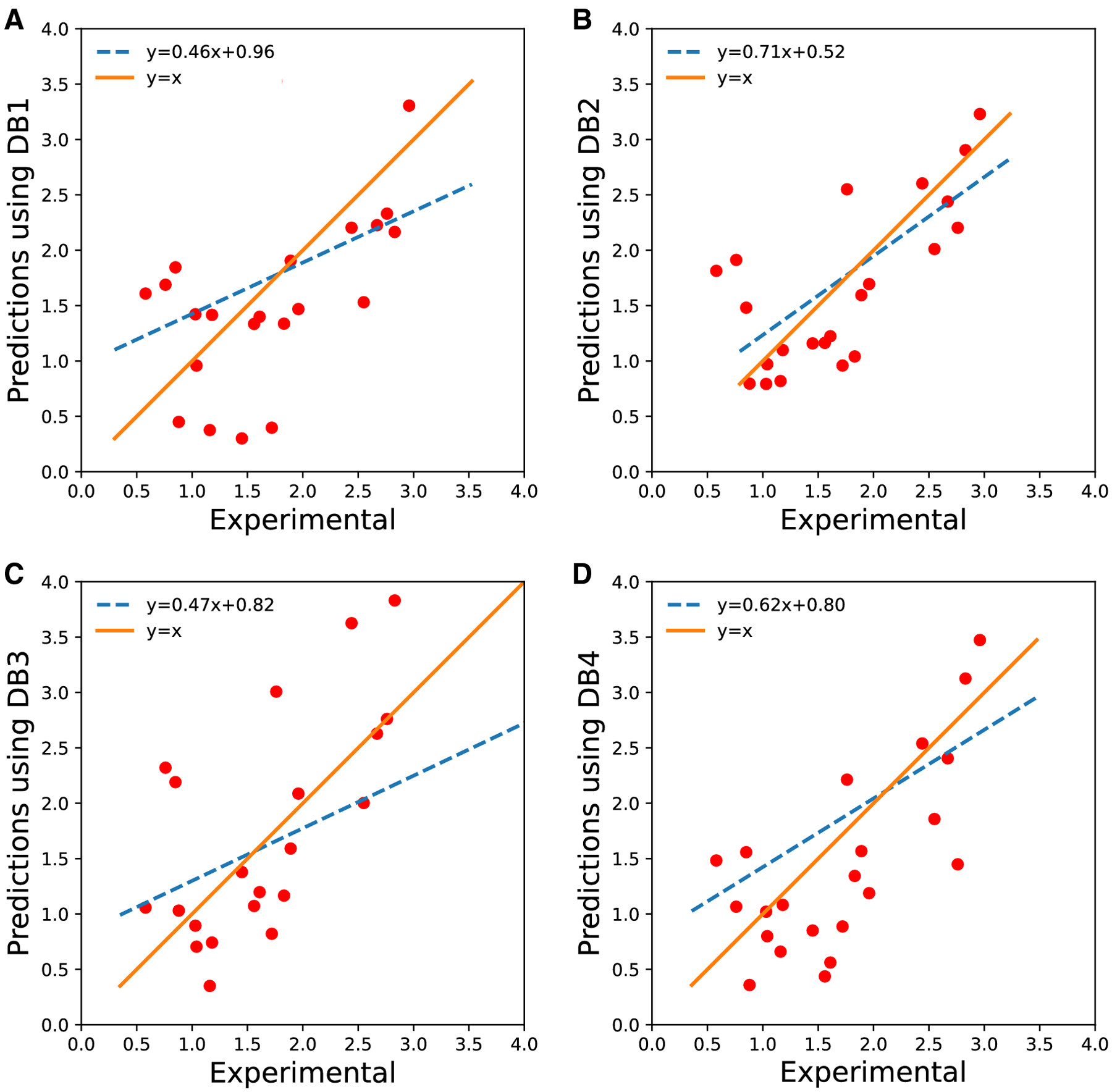
The best fit lines for prediction sets. The experimental versus prediction values are shown in red circles as a scatter plot. The actual fit line is shown in orange. The dashed blue curve shows the best fit line. A) predictions using DB1, B) predictions using DB2, C) predictions using DB3, and D) predictions using the DB4 training set.

Four sets of blind predictions were generated using different training sets, as described above. By the rules of the SAMPL7 challenge, only one of these predictions could be used as a “ranked” submission. To determine which set to use we examined the performance of each predictor on the FDA [8] and Huuskonen test sets [14] and chose the model with the lowest RMSE: ClassicalGSG-DB3. We note that there was an error in our analysis code at this time, and later we found the ClassicalGSG-DB1 was the model with the lowest RMSE. The RMSE and *r*^2^ values of prediction sets using these four models was determined after the SAMPL7 challenge (Table 4). Note that the best model in terms of RMSE is the one trained on the DB2 training set and the worst performing model was DB3. The model we were intending to select, DB1, is the second-worst performing model. In retrospect, this shows that the FDA and Huuskonen test sets were not good proxies for the SAMPL7 molecules. We show comparisons of our predictions with experimental results, along with linear fit lines for each model in Figure 4. Note the best fitting coefficients also correspond to the model trained on the DB2 training set.

**Fig. 5:**
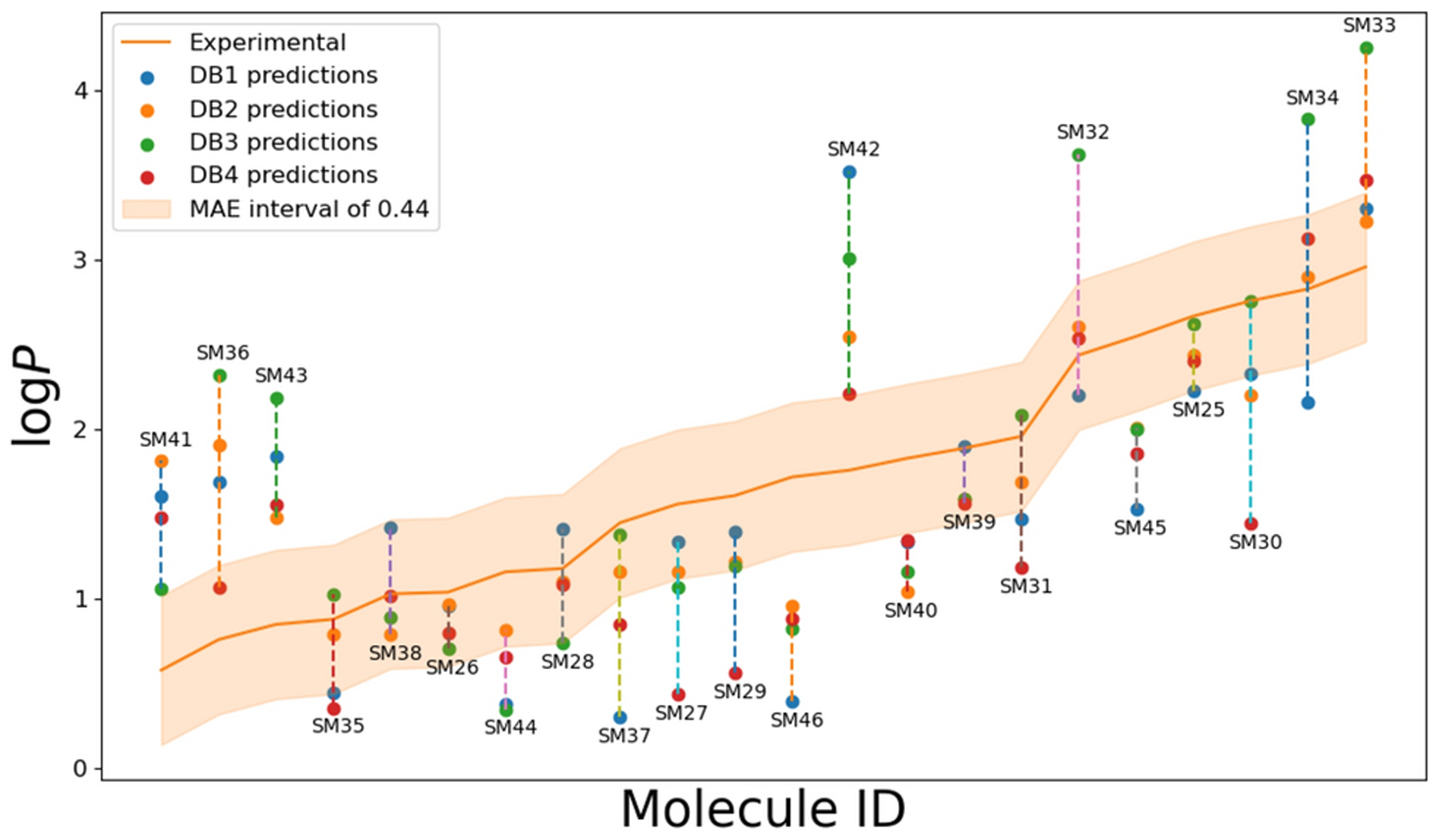
The log *P* predictions from our submissions to the SAMPL7 challenge. The orange line shows the experimental values. The ClassicalGSG predictions are shown as circles (DB1: blue, DB2: orange, DB3: green, DB4: red). The thick orange area shows the MAE interval of 0.44, which is the lowest MAE of our submitted predictions (ClassicalGSG-DB2). Molecules are labeled with their molecule ID from SAMPL7 [27].

To identify the prediction outliers, we show the log *P* predictions from our methods for the SAMPL7 target molecules in Figure 5. We find the largest systematic errors in compounds: SM36, SM40, SM41, SM42, SM43 and SM45 molecules. As shown in Figure 6A from Ref. [27], these molecules were found to have some of the highest prediction errors across all submissions. For SM36, SM41, SM42 and SM43, ClassicalGSG consistently over-predicted the experimentally determined log *P*, which was also true for the other well-performing methods (in Figure 6D of Ref. [27]). Molecules SM40 and SM45 were underpredicted compared to experiment, which was also in line with other well-performing methods, although the trend is less clear.

### Performance of Open-source force fields

**Fig. 6:**
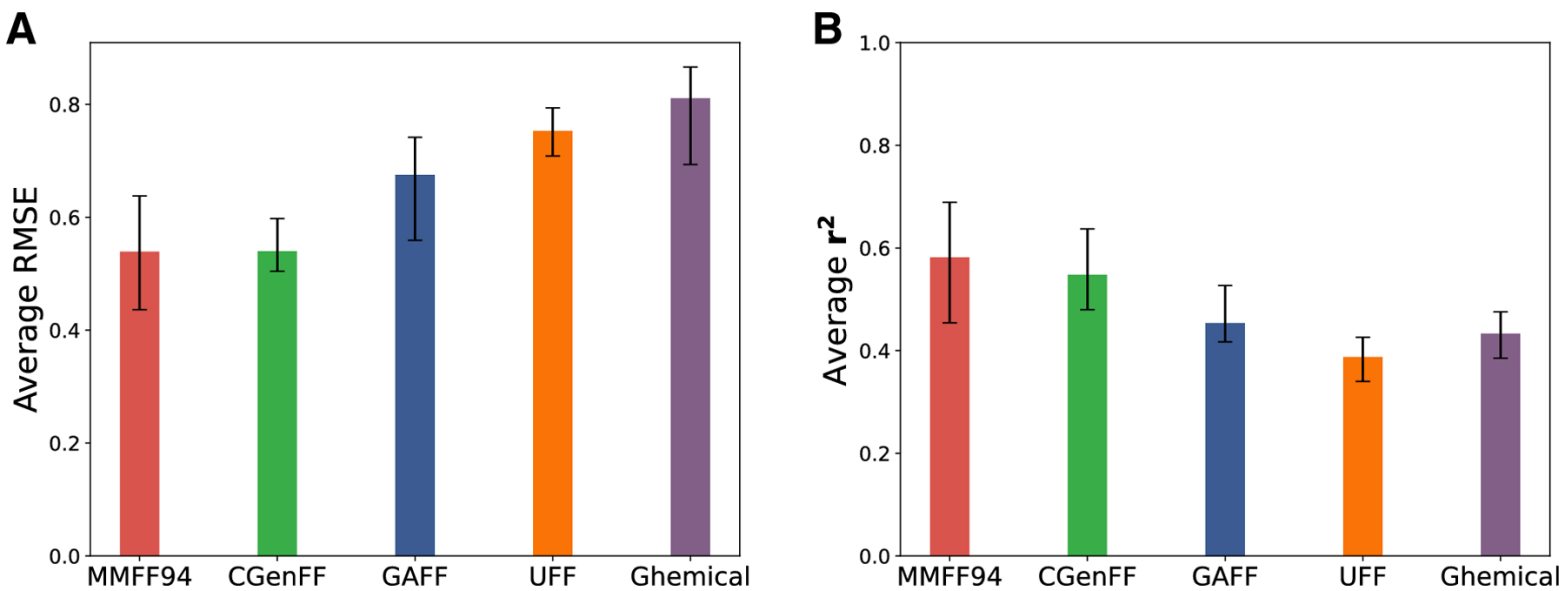
Results of ClassicalGSG models trained using open-source force field parameters. Error bars are computed over five independently-trained models. These models are trained using the 2D structure information and using all the scattering moments with the maximum wavelet scale (*J*) of 4. For each set of ClassicalGSG models trained using these force field parameters we show A) the average RMSE, and B) the average *r*^2^.

Although there is an online server for generating CGenFF parameter files for a given molecule, it is still challenging to use CGenFF for high throughput applications as it is not open-source. Hence, we decided to assess the performance of open-source force field tools implemented by OpenBabel [69, 70] which is open-source and free to use on large databases of molecules. We utilized GAFF [58], UFF [59], MMFF94 [60, 61] and Ghemical [62] force field parameters to generate atomic attributes including atom types, partial charges and the two Lennard-Jones interaction parameters (*ϵ* and *r*). As above, we applied the GSG method with maximum wavelet scale of 4 while using all scattering operators to generate molecular features from atomic attributes. We used the DB2 training set to train 5 log *P* predictor models for each force field. Each of these models is trained using a 5-fold cross validation approach. These models are tested on the SAMPL7 molecules and the RMSE and *r*^2^ values are calculated for each set of predictions. We took the average values over the 5 runs for each force field and the results are shown in Figure 6. This figure shows that ClassicalGSG models from MMFF94 force field parameters achieve the highest *r*^2^ and lowest RMSE value, which are on par with the CGenFF results submitted to the challenge.

Additionally, we studied the performance of MMFF94 ClassicalGSG models on independent external test sets such as FDA [8], Huuskonen [14, 66], Star [17], NonStar [17] and the compounds from the SAMPL6 log *P* prediction challenge [26]. For the purpose of a fair comparison, we used the same 10722 molecules from the OpenChem dataset as utilized in our previous paper [24]. All combinations of a set of maximum wavelet scales (*J*) and sets of scattering operators are used as GSG parameters to train 20 ClassicalGSG models as indicated in Table 5. The atomic attributes were generated from MMFF94 atomic parameters and all atomic types from MMFF94 (ACall). The parameters corresponding to the best models per each test test along with their performance results are shown in Table 6. The ClassicalGSG models based on MMFF94 achieve better performance compared to CGenFF based models for all test sets (see Table 7 in Ref. [24]). The comparison between log *P* prediction results for FDA, Star, and NonStar test sets and those from other log *P* predictor methods are shown in Tables S1, S2, and S3. As these tables show, MMFF94 ClassicalGSG achieves the best results to date for the NonStar test set and the second-best results for the FDA and Star test sets. Moreover, our method shows a significant improvement in the prediction of log *P* values for SAMPL6 molecules, with a RMSE in the range [0.29, 0.52] and median of 0.42 over 20 models. This compares favorably to the best performing model (Cosmotherm [36]) with an RMSE of 0.35 in the SAMPL6 blind challenge.

## 4 Discussion and Conclusions

In this work, we described the curation of four training sets that we utilized to train ClassicalGSG log *P* predictor models for the SAMPL7 physical property blind challenge. The molecular features originally submitted for these models were created by CGenFF force field parameters. Our most accurate set of predictions – with an RMSE of 0.55 and MAE of 0.44 – were made by the ClassicalGSG-DB2 model, which had the lowest RMSE among the 36 submitted sets of predictions based on the non-ranked predictions analysis. Our ranked predictions were from ClassicalGSG-DB3 – with an RMSE of 0.77 and MAE of 0.62 – which were ranked in 5th place. To further compare ClassicalGSG with other predictors we also made post-hoc predictions for the previous SAMPL6 challenge molecules. We trained 20 predictors using different parameters and obtained some estimates that had significantly lower RMSE (0.29) than the best performing model at the time (Cosmotherm [36], 0.35). However, this parameter selection had the benefit of hindsight, so a more meaningful comparison is with our median RMSE of 0.42. We note that this RMSE would have placed fourth among the submissions to SAMPL6 [26].

Here we trained several ClassicalGSG models on molecular features generated by atomic attributes from open-source force fields. We find that MMFF94 ClassicalGSG models are slightly more accurate than CGenFF ClassicalGSG models. Applying the MMFF94 log *P* predictor model trained on the OpenChem dataset to external test sets obtains excellent results throughout, at times achieving the best results acquired to date. An added benefit is that the MMFF94 ClassicalGSG models provide an end-to-end framework for predicting log *P* values using only SMILES strings as input and does not require any auxiliary stream files like the CGenFF models. It might be counter-intuitive that a force field developed in the 1990s [60, 61] would outperform a modern forcefield that is still being actively developed [54, 55]. We note here that pertinent features of force fields for predicting log *P* values are very different from those needed to conduct physically-meaningful molecular dynamics simulations. We suspect that the leading benefit of MMFF94 is its broad coverage of atom types describing the chemical features of small, organic molecules that are relevant to log *P*. Differences in one-hot encodings of atom type would likely have a much stronger impact than improving predictions of partial charges, for example.

Our code is publicly available on GitHub https://github.com/ADicksonLab/ClassicalGSG and our training and test sets are available in SDF format on Zenodo https://doi.org/10.5281/zenodo.4560967. The ClassicalGSG repository contains two pre-trained log *P* predictors, one using MMFF94 and another one using CGenFF atomic attributes. Once the predictor is trained, values can be predicted extremely quickly. Predictions for a set of 1000 molecules can be made in about 150 seconds on an Intel i7 processor, without parallelization. The code provides modules for extracting GSG features and training NN models on new datasets.

As mentioned in our previous work [24] the ClassicalGSG method is not specific to log *P* and could predict other molecular properties as well. We emphasize that progress in the field of molecular property prediction can be greatly accelerated by the free sharing of molecular property datasets. Efforts such as OpenChem [23] that support the sharing of methods and datasets will be useful catalysts for methods development. Publicly available data sources for properties such as intestinal permeability, pKa values, intrinsic clearance rates (CL_int_) and serum protein binding fractions would similarly be great catalysts for the development of accurate predictors of pharmacokinetic effects.

## Supplementary Material

1720201_Supp_info

## Figures and Tables

**Table 1:** log *P* datasets used for training.

Test set name	Number of molecules
PHYSPROP [65]	41039
Huuskonen training set [14, 66]	1496
TopP-S [14]	8199
OpenChem [23]	14176
ALOGPS_3_01	17436
Logpt_all_data_training	233
Logpt_challenge_training	187

**Table 2:** The parameters hyperparameters of neural networks. Sets in square brackets denote possible parameter values used in the grid search method.

Parameter	Values
Number of hidden layers	[2, 3, 4, 5]
Size of hidden layers	[300, 400, 500]
Dropout rate	[0.2, 0.4]
Initial learning rate	0.005
Learning coefficient	0.5
Batch size	256
Max epoch size	400

**Table 3:** The PIs and coverage range for the SAMPL7 molecules using four ClassicalGSG methods. $\hat{y}$ is the prediction of log *P*.

Model name	PIs	Coverage of PIs
ClassicalGSG_DB1	$\hat{y}$ ± 0.94	72.72%
ClassicalGSG_DB2	$\hat{y}$ ± 0.88	90.90%
ClassicalGSG_DB3	$\hat{y}$ ± 0.88	68.18%
ClassicalGSG_DB4	$\hat{y}$ ± 0.98	86.36%

**Table 4:** The log *P* prediction results for the SAMPL7 molecules.

Model name	RMSE	*r*^2^	MAE	Ranking among all verified predictions	Ranking among ranked predictions
ClassicalGSG_DB2	0.55	0.51	0.44	1	
ClassicalGSG_DB4	0.65	0.50	0.56	3	
ClassicalGSG_DB1	0.76	0.28	0.62	7	
ClassicalGSG_DB3	0.77	0.51	0.62	9	5

**Table 5:** Sets of parameters used to evaluate MMFF94 ClassicalGSG models on external test sets. Sets in square brackets denote all the GSG parameter values used for generating the molecular features. For scattering operators, “z” denotes the zeroth order operator (Equation 3), “f” is first order (Equation 4), and “s” is second order (Equation 5).

Parameter	Values
Max. wavelet scale (*J*)	[4, 5, 6, 7, 8]
Scattering operators	[(z, f), (z, s), (f, s), (z, f, s)]

**Table 6:** The logP prediction results from MMFF94 force field parameters for external test set.

GSG parameters		Performance results			
Max. wavelet scale	Scattering operators	Test set name	RMSE	*r*^2^	MAE
7	(f,s)	FDA	0.53	0.93	0.27
7	(z,f,s)	Star	0.44	0.93	0.29
5	(z,f)	NonStar	0.74	0.89	0.59
7	(z,f)	Huuskonen	0.35	0.94	0.18
5	(Z,f,s)	SAMPL6	0.29	0.87	0.23
