# Supplementary material for: Predicting partition coefficients for the SAMPL7 physical property challenge using the ClassicalGSG method
Source: J Comput Aided Mol Des. Author manuscript; Available in PMC 2022 Jul 1. (PMC8295205; doi:10.1007/s10822-021-00400-x)
Supplement: 1720201_Supp_info [file NIHMS1720201-supplement-1720201_Supp_info.pdf]

Predicting partition coefficients for the SAMPL7 physical property challenge using the ClassicalGSG method: Supplemental Information

Nazanin Donyapour · Alex Dickson

Table 1 The $\log P$ prediction results from different $\log P$ methods for the FDA test set. The TopP-S is the best performing method (GBDT-ESTD^{+}-2-AD) on FDA test set from [1]. Table from [1].

FDA test set, N=406<br>2 molecules are removed due to pre-processing failure			
Method	r^2	RMSE	MAE
TopP-S	0.935	0.51	0.24
MMFF94-ClassicalGSG	0.929	0.535	0.27
CGenFF-ClassicalGSG	0.910	0.55	0.35
ALOGPS	0.908	0.6	0.42
XLOGP3	0.872	0.72	0.51
XLOGP3-AA	0.847	0.8	0.57
CLOGP	0.838	0.88	0.51
TOPKAT	0.815	0.88	0.56
ALOGP98	0.800	0.90	0.64
KowWIN	0.771	1.10	0.63
HINT	0.491	1.93	1.3

N. Donyapour · A. Dickson
Department of Computational Mathematics, Science and Engineering, Michigan State University, East Lansing, Michigan, USA

A. Dickson
Department of Biochemistry and Molecular Biology, Michigan State University, East Lansing, Michigan, USA E-mail: alexrd@msu.edu

Table 2 The $\log P$ prediction results from different $\log P$ methods for the Star test set. The TopP-S is the best performing method (MT-ESTD$^+$-1-AD) on Star test set from [1]. Table from [1].

Star test set, N=223				
		% of molecules within error range		
Method	RMSE	< 0.5	1 <	1 >
AB/LogP	0.41	84	12	4
MMFF94-ClassicalGSG	0.44	81	14	5
S+logP	0.45	76	22	3
TopP-S	0.49	77	16	7
CGenFF-ClassicalGSG	0.49	75	18	7
ACD/logP	0.5	75	17	7
CLOGP	0.52	74	20	6
VLOGP OPS	0.52	64	21	7
ALOGPS	0.53	71	23	6
XLOGP3	0.62	60	30	10
KowWIN	0.64	68	21	11
CSLogP	0.65	66	22	12
ALOGP	0.69	60	25	16
MolLogP	0.69	61	25	14
ALOGP98	0.7	61	26	13
OsirisP	0.71	59	26	16
VLOGP	0.72	65	22	14
TLOGP	0.74	67	16	13
ABSOLV	0.75	53	30	17
QikProp	0.77	53	30	17
QuantlogP	0.80	47	30	22
SLIPPER-2002	0.80	62	22	15
COSMOFrag	0.84	48	26	19
XLOGP2	0.87	57	22	20
QLOGP	0.96	48	26	25
VEGA	1.04	47	27	26
CLIP	1.05	41	25	30
LSER	1.07	44	26	30
MLOGP(Sim+)	1.26	38	30	33
NC+NHET	1.35	29	26	45
SPARC	1.36	45	22	32
HINTLOGP	1.80	34	22	44

Table 3 The log P prediction results from different log P methods for the NonStar test set. The TopP-S is the best performing method (MT-ESTD$^+$-2-AD) on NonStar test set from [1]. Table from [1]

NonStar test set, N=43				
		% of molecules within error range		
Method	RMSE	< 0.5	1 <	1 >
MMFF94-ClassicalGSG	0.74	49	35	16
ALOGPS	0.82	42	30	28
MiLogP	0.86	49	30	21
S+logP	0.87	40	35	26
XLOGP3	0.89	47	23	30
CLOGP	0.91	47	28	26
ALOGP	0.92	28	40	33
CSLogP	0.93	58	19	23
MolLogP	0.93	40	25	26
TopP-S	0.94	51	19	30
OsirisP	0.94	42	26	33
ALOGP98	1.00	30	37	33
AB/LogP	1.00	42	23	35
ACD/logP	1.00	44	32	23
CGenFF-ClassicalGSG	1.02	36	31	33
ABSOLV	1.02	49	28	23
KowWIN	1.05	40	30	30
VLOGP OPS	1.07	33	28	26
TLOGP	1.12	30	37	30
VLOGP	1.13	40	28	33
XLOGP2	1.16	35	23	42
SLIPPER-2002	1.16	35	23	42
QuantlogP	1.17	35	26	40
COSMOFrag	1.23	26	40	23
VEGA	1.24	28	30	42
QikProp	1.24	40	26	35
LSER	1.26	35	16	49
QLOGP	1.42	21	26	53
CLIP	1.54	33	9	49
MLOGP(Sim+)	1.56	26	28	47
SPARC	1.70	28	21	49
NC+NHET	1.71	19	16	65
HINTLOGP	2.72	30	5	65

References

1. K. Wu, Z. Zhao, R. Wang, G.W. Wei, Journal of computational chemistry **39**(20), 1444 (2018)
